# Comparative Analysis of Antithrombotic Strategies Following Valve-in-Valve Transcatheter Aortic Valve Implantation Using Real-World Registry Data

**DOI:** 10.1007/s10557-026-07914-2

**Published:** 2026-07-17

**Authors:** Edward Dababneh, Maria Gabriela Matta, Ajay Sinhal, Selvanayagam Niranjan, Sylvio Provenzano, Anthony Camuglia, Ian Hughes, Kuljit Singh

**Affiliations:** 1 Department of Cardiology, Gold Coast Health Service, Gold Coast, QLD Australia; 2 Department of Cardiology, Flinders Medical Centre, Adelaide, South Australia Australia; 3 Department of Medicine, Griffith University School of Medicine, Gold Coast, QLD Australia; 4 Department of Medicine, Bond University, Gold Coast, QLD Australia; 5 Department of Cardiology, Princess Alexandra Hospital, Woolangabba, QLD Australia; 6 Department of Medicine, University of Queensland Brisbane, Brisbane, QLD Australia; 7 Department of Medicine, University of Adelaide, Adelaide, SA Australia; 8 Gold Coast University Hospital, Griffith University, 1 Hospital Blvd, Southport, QLD 4215 Australia

**Keywords:** ViV TAVI, Antithrombotic, Oral anticoagulation

## Abstract

**Background:**

Valve-in-valve transcatheter aortic valve implantation is a less invasive alternative to redo surgery for failed surgical bioprostheses, but optimal antithrombotic management is uncertain.

**Objectives:**

To compare outcomes across antithrombotic regimens after valve-in-valve procedures in real-world practice.

**Methods:**

In the Australasian Cardiac Outcomes Registry (2018–2025), 1,050 adults were grouped by discharge regimen: single antiplatelet therapy, dual antiplatelet therapy, oral anticoagulation, or oral anticoagulation plus antiplatelet therapy. Outcomes included 30-day and 12-month mortality, stroke/TIA, life-threatening or major bleeding, major adverse cardiac and cerebrovascular events, and echocardiographic valve performance.

**Results:**

Thirty-day mortality was higher with oral anticoagulation (1.7%) and combination therapy (3.6%) than with single or dual antiplatelet therapy (both 0%; *p* < 0.001). The 30-day MACCE rate was greater with oral anticoagulation (4.5%) and combination therapy (4.4%) than with single (1.5%) or dual antiplatelet therapy (0.4%) (*p* = 0.003); stroke/TIA and bleeding were infrequent and similar across groups. At 12 months, MACCE remained highest with oral anticoagulation (11.8%) and combination therapy (10.7%) compared with single (5.0%) and dual antiplatelet therapy (3.2%) (*p* = 0.001). Mortality at 12 months was numerically higher with oral anticoagulation or combination therapy (5.2% and 4.4%) than with single or dual antiplatelet therapy (1.8% and 2.4%) (*p* = 0.116). At 30 days, transvalvular gradients were lower in patients receiving oral anticoagulation-containing regimens; however, no significant differences in gradients, valve area, ejection fraction, or regurgitation were observed at 12 months. In multivariable analysis, combination therapy was independently associated with higher odds of 12-month MACCE compared with SAPT (OR 2.76, 95% CI 1.03–7.37; *p* = 0.043). Higher STS score and greater frailty were also independently associated with 12-month MACCE.

**Conclusions:**

In this observational cohort of patients undergoing valve-in-valve TAVI, oral anticoagulation-containing regimens were associated with higher early mortality and higher 12-month MACCE rates than antiplatelet-only strategies. However, these associations should be interpreted cautiously, as they likely reflect in part the greater baseline comorbidity burden of patients prescribed anticoagulation rather than a direct treatment effect. No sustained haemodynamic benefit was observed at 12 months. Individualised antithrombotic therapy and prospective randomised studies are warranted.

**Supplementary Information:**

The online version contains supplementary material available at https://doi.org/10.1007/s10557-026-07914-2.

## Introduction

Valve-in-valve (ViV) transcatheter aortic valve implantation (TAVI) is an increasingly common procedure used to treat failed surgical bioprosthetic aortic valves, especially in high-risk or inoperable patients. ViV TAVI offers a less invasive alternative to repeat surgery with favourable outcomes in terms of symptom relief and procedural safety [1]. However, this approach presents unique haemodynamic and anatomic challenges due to the constrained space within the surgical valve frame, leading to higher gradients, an increased risk of acute and subacute coronary obstruction, and a greater risk of subclinical leaflet thrombosis [2]. 

TAVI valve thrombosis—either clinical or subclinical—has emerged as a concern, potentially compromising valve function and durability [3]. The PARTNER 3 trial found that valve thrombosis, at 2 years, was significantly higher in patients who underwent TAVR than in those who underwent surgical replacement (2.6% vs. 0.7%) [4]. The rate of clinical valve thrombosis and bioprosthetic valve degeneration is approximately 1% to 3% after TAVI, while subclinical valve thrombosis is found in about 10% to 15% of patients at 30 days [5–7]. In one observational study, the median time for TAVI thrombosis was 180 days, and valve thrombosis in ViV TAVI was approximately six times higher than in native-valve TAVI [5]. 

Antithrombotic therapy following ViV TAVI is complex and remains an area of ongoing investigation. Patients undergoing ViV procedures often have both high thrombotic and bleeding risks, because of the underlying comorbidities such as atrial fibrillation, kidney disease, anaemia, heart failure and prior antiplatelet and anticoagulant use [8]. Oral anticoagulation (OAC) has been found to reduce the risk of TAVI thrombosis but comes at the inherent risk of significant bleeding [5]. Both bleeding and thrombosis lead to high mortality. Current practice extrapolates recommendations from native-valve TAVI, where dual antiplatelet therapy (DAPT) or single antiplatelet therapy (SAPT) is used, often guided by bleeding risk unless the patient is on OAC for other reasons [7]. A higher incidence of leaflet thrombosis has been observed in ViV TAVI patients, prompting debate over whether OAC may offer greater benefit in this subgroup. Also of note is that while the GALILEO and ATLANTIS trials provided important insights into antithrombotic therapy after native-valve TAVI, their findings are not directly generalisable to ViV TAVI populations, where the thrombotic milieu and haemodynamic environment differ substantially from native-valve procedures [9, 10]. 

While ViV-specific evidence is beginning to emerge, including a 2025 JACC: Cardiovascular Interventions report suggesting a possible reduction in 1-year stroke incidence with DAPT over SAPT after ViV-TAVR, the field remains underdeveloped and real-world comparative data are limited [11]. The present study contributes one of the largest real-world registry datasets to this evolving evidence base, utilising data from the Australasian Cardiac Outcomes Registry (ACOR) TAVI Registry to evaluate the comparative effectiveness and safety of different antithrombotic strategies following ViV TAVI.

The primary hypothesis of this study was that discharge antithrombotic regimen would be associated with differences in clinical outcomes and valve haemodynamics at 30 days and 12 months following ViV TAVI. Accordingly, we evaluated the incidence of all-cause mortality, stroke/TIA, bleeding, and major adverse cardiac and cerebrovascular events (MACCE), together with valve haemodynamic performance assessed by transthoracic echocardiography, among patients receiving different antithrombotic regimens after ViV TAVI.

## Methods

### Study Design and Data Source

This was a cohort study using data from the ACOR TAVI Registry, a nationwide prospective clinical registry established in 2018 to monitor outcomes and procedural quality of TAVI in Australia. Managed by the South Australian Health and Medical Research Institute, the Registry supports clinical governance and reimbursement pathways for participating centres. Data collection is facilitated by local TAVI coordinators and standardised across centres using Valve Academic Research Consortium (VARC-3) definitions for procedural and clinical outcomes [12, 13]. 

All registry data are entered into a secure, centralised Research Electronic Data Capture (REDCap) system and are governed by a national steering committee. Patients are included in the registry via an opt-out consent process approved by the institutional ethics board (HREC Reference No: HREC/18/SAC/67, 2017, Adelaide, SA, Australia). This study was deemed to be research not requiring ethical review by the Gold Coast Hospital and Health Service (Study ID #112933) (HREC/2025/QGC/112933).

### Study Population

The analysis included adult patients who underwent ViV TAVI for treatment of degenerated bioprosthetic aortic valves. Inclusion criteria required documentation of a ViV TAVI procedure in the registry and availability of discharge antithrombotic therapy and primary clinical outcome data. Echocardiographic follow-up was collected when available. Patients were excluded if they had incomplete information on antithrombotic therapy at discharge.

### Antithrombotic Therapy Classification

Patients were categorised based on their discharge antithrombotic regimen into four groups:

1. Single antiplatelet therapy – aspirin monotherapy.
2. Dual antiplatelet therapy – aspirin in combination with clopidogrel or ticagrelor.
3. Anticoagulation alone – either vitamin K antagonists or direct oral anticoagulants.
4. Combination therapy – anticoagulation with concurrent antiplatelet therapy.

### Clinical Outcomes

MACCE was defined as a composite of major vascular complications, myocardial infarction, cerebrovascular accident (CVA), major cardiac complications or death. Major cardiac complications were defined as cardiac tamponade, device embolisation necessitating an additional intervention, and annular rupture [12]. 

Bleeding was defined as either life-threatening or disabling bleeding – (Fatal bleeding OR - Bleeding in a critical area or organ, such as intracranial, intraspinal, intraocular, or pericardial necessitating pericardiocentesis, or intramuscular with compartment syndrome OR - Bleeding causing hypovolaemic shock or severe hypotension requiring vasopressors or surgery OR - Overt source of bleeding with drop in haemoglobin of 5 g/dl or whole blood or packed red blood cells (RBCs) transfusion 4 Units) or Major bleeding – (Overt bleeding either associated with a drop in the haemoglobin level of at least 3.0 g/dl or requiring transfusion of two or three units of whole blood/ RBC AND - Does not meet criteria of life-threatening or disabling bleeding).

Stroke/TIA was defined as any of the following: transient ischemic attack (TIA), haemorrhagic stroke, ischaemic stroke, or undetermined stroke in which a neurologist or neurosurgeon confirmed the diagnosis.

### Data Collection and Management

Key clinical, procedural, and echocardiographic variables were collected prospectively at baseline, discharge, and follow-up intervals. All outcomes were adjudicated by site specific coordinators according to VARC-3 criteria; no independent central adjudication committee was used. De-identified data extracts were provided for analysis upon approval by the ACOR TAVI Research & Publications Committee.

### Statistics

The frequency and distribution of variables were initially examined both overall and stratified by antithrombotic regimen. Continuous variables with a normal distribution are presented as mean ± standard deviation, while non-normally distributed continuous variables are summarised using medians and interquartile ranges. Categorical variables are reported as counts and percentages.

Differences in mean aortic valve gradients among antithrombotic groups at 30 days and 12 months were assessed using one-way analysis of variance (ANOVA). When ANOVA indicated a significant overall difference, pairwise comparisons were performed with Bonferroni correction to adjust for multiple testing. Homogeneity of variances was verified using Bartlett’s test.

Categorical outcomes, including bleeding and stroke/TIA events, were compared across antithrombotic groups using Fisher’s exact test due to the low frequency of events. Because clinical outcomes in the ACOR TAVI Registry were captured at predefined follow-up intervals (30 days and 12 months) and exact event dates were not available in the registry extract, time-to-event analyses could not be reliably performed. Accordingly, outcomes were analysed as binary events at fixed follow-up time points.

Multivariable logistic regression models were used to estimate odds ratios (ORs) and 95% confidence intervals (CIs) for clinical outcomes across antithrombotic regimens, using SAPT as the reference group. Covariates were selected a priori based on their established clinical relevance and potential confounding effects and included age, Society of Thoracic Surgeons risk score (STS), Rockwood Clinical Frailty Scale, prior percutaneous coronary intervention (PCI), atrial fibrillation, and prior stroke/TIA. To assess whether the association between antithrombotic regimen and MACCE differed according to atrial fibrillation status, an interaction term between antithrombotic treatment group and baseline atrial fibrillation was evaluated in the multivariable model. To assess the robustness of the findings, a sensitivity analysis using a more parsimonious adjustment model including only age, STS score, and Rockwood Clinical Frailty Scale was performed. Model performance was assessed using likelihood ratio chi-square tests, Pseudo R² statistics, the Hosmer–Lemeshow goodness-of-fit test for calibration, and the area under the receiver operating characteristic curve (C-statistic) for discrimination.

Inverse Probability Weighted Regression Adjustment (IPWRA) was used to estimate treatment effects while accounting for baseline differences between antithrombotic groups. For echocardiographic outcomes, IPWRA was applied to evaluate the effect of treatment on left ventricular ejection fraction, aortic valve area, peak velocity, and mean transvalvular gradient at 12 months. This analysis included only patients with echocardiographic measurements available at both 30 days and 12 months. Models were adjusted for the corresponding 30-day post-procedure echocardiographic value, as well as age, prior PCI, atrial fibrillation, and prior stroke/TIA.

As a sensitivity analysis for clinical outcomes, IPWRA was also performed for 12-month MACCE to address potential confounding by indication. The treatment model included baseline variables considered likely to influence antithrombotic treatment selection, including chronic kidney disease, prior acute myocardial infarction, atrial fibrillation, prior stroke, prior PCI, and prior coronary artery bypass graft surgery (CABG). The outcome model included clinically relevant predictors of adverse cardiovascular outcomes, including coronary artery disease, diabetes mellitus, chronic kidney disease, STS score, Rockwood Clinical Frailty Scale, and prior CABG.

All statistical analyses were two-tailed, with a significance threshold of *p* < 0.05. Analyses were performed using Stata Statistical Software, version 19.0 (StataCorp, 2025).

## Results

### Basic Characteristics

A total of 1,050 patients underwent ViV TAVI, of which 269 received aspirin monotherapy, 243 aspirin plus P2Y12 inhibitor, 289 were on OAC, and 249 were on combination therapy, which consisted of concomitant oral anticoagulation with single antiplatelet. The mean age ranged from 76.9 to 79.4 years, with a predominance of male patients across all groups. Hypertension was the most common comorbidity, followed by diabetes. As expected, atrial fibrillation was highly prevalent in the anticoagulant (74.4%) and combination therapy (52.2%) groups. Coronary artery disease and prior PCI were more common in the DAPT group. Heart failure and chronic kidney disease were frequent, particularly in the anticoagulant group (Table 1). Most patients had NYHA class II–III symptoms. The median Rockwood Clinical Frailty Scale was 4 across all groups; however, median STS score was highest in the OAC and combination therapy groups.

**Table 1:** Baseline characteristics

	Single antiplatelet (*n* = 269)	Dual antiplatelet (*n* = 243)	Anticoagulation (*n* = 289)	Combination therapy (*n* = 249)	*P* value
Demographic					
Age (years), mean (SD)	76.9 (10.0)	78.9 (8.7)	79.4 (7.8)	78.4 (10.5)	0.009
Male sex, n (%)	179 (66.5)	166 (68.3)	181 (62.6)	165 (66.3)	0.560
Comorbidities, n (%)					
Hypertension	195 (72.5)	186 (76.5)	211 (73.0)	177 (71.4)	0.598
Diabetes	73 (27.3)	60 (24.7)	63 (21.8)	59 (23.7)	0.495
Known Atrial Fibrillation	39 (14.6)	25 (10.3)	215 (74.4)	130 (52.2)	< 0.001
Coronary Artery Disease	154 (57.5)	162 (66.9)	157 (54.7)	156 (62.9)	0.020
Prior PCI	21 (7.8)	57 (23.6)	23 (8.0)	60 (24.5)	< 0.001
Congestive Cardiac Failure	102 (38.2)	131 (53.9)	146 (50.5)	120 (48.2)	0.002
Prior Stroke	9 (3.3)	18 (7.4)	33 (11.4)	21 (8.4)	0.005
Peripheral Arterial Disease	36 (13.4)	35 (14.4)	26 (9.1)	36 (14.5)	0.181
Current/Recent Smoker	12 (4.5)	6 (2.5)	6 (2.1)	5 (2.0)	0.258
Chronic Kidney Disease	98 (36.6)	102 (42.0)	130 (45.1)	106 (42.6)	0.222
Dialysis	4 (1.5)	6 (2.5)	1 (0.3)	5 (2.0)	0.210
Clinical Scores					
NYHA I, n (%)	2 (0.8)	3 (1.3)	1 (0.4)	3 (1.3)	-
NYHA II, n (%)	101 (40.2)	97 (40.6)	86 (31.0)	87 (36.4)	0.739
NYHA III, n (%)	126 (50.2)	117 (49.0)	152 (54.9)	111 (46.4)	0.854
NYHA IV, n (%)	22 (8.8)	22 (9.2)	38 (13.7)	38 (15.9)	0.773
STS Score,, median (IQR)	4.57 (2.76–7.88)	4.97(3.02–8.35)	6.00 (4.00–9.82)	6.00 (3.70–10.70)	< 0.001
Rockwood Clinical Frailty Scale (median)	4	4	4	4	0.305
Investigations					
Hemoglobin, mean (SD), g/L	124.7 (17.4)	123.4 (17.3)	122.8 9 (17.6)	122.4 (18.4)	0.342
Creatinine, median (IQR), micrl/L	94 (77.5–116)	101 (80–130)	102.5 (80–131)	100.5 (81–130.5)	0.172
Albumin, mean (SD), g/L	38.7 (5.4)	37.2 (4.9)	37.4 (5.3)	37.4 (5.1)	0.016
Medications, n (%)					
ACE/ARB	135 (50.2)	123 (50.6)	136 (47.1)	123 (49.4)	0.841
Beta Blocker	99 (36.8)	78 (32.1)	126 (43.6)	108 (43.4)	0.019
Statin	169 (62.8)	154 (63.4)	174 (60.2)	137 (55.0)	0.206
Diuretic	104 (38.7)	103 (42.4)	162 (56.1)	124 (49.8)	< 0.001
Indication, n (%)					0.557
Predominant Stenosis	124 (46.4)	98 (40.3)	138 (47.8)	119 (47.8)	
Predominant Incompetence	69 (25.8)	78 (32.1)	91 (31.5)	61 (24.5)	
Mixed	73 (27.3)	65 (26.7)	60 (20.8)	66 (26.5)	
No data	3 (1.1)	2 (0.8)	0 (0.0)	3 (1.2)	
Baseline Echocardiography					
Ejection Fraction, mean (SD), %	56 (11.6)	56 (12.6)	54 (11.8)	54 (13.3)	0.132
LV Internal Diameter Syst,, mean (SD), cm	3.6 (1.2)	3.5 (0.9)	3.6 (0.9)	3.4 (0.9)	0.221
LV Internal Diameter Dias, mean (SD), cm	5.1 (1.0)	5.2 (0.9)	5.1 (0.8)	5.0 (0.8)	0.516
Aortic Valve Peak Velocity, median (IQR), m/s	4.0 (3.1–4.5)	3.9 (3.1–4.3)	3.72 (2.9–4.3)	3.9 (3.02–4.39)	0.191
Aortic Valve Mean Gradient, median (IQR), mmHg	38 (22–47)	34 (21–45)	33 (19–45)	36 (21–46)	0.123
Aortic Valve Area, median (IQR), cm^2^	1.0 (0.8–1.4)	1.0 (0.7–1.4)	1.09 (0.8–1.5)	1.0 (0.8–1.4)	0.573
Aortic Valve Dimensionless Index, median (IQR)	0.28 (0.22–0.40)	0.30 (0.22–0.41)	0.29 (0.23–0.38)	0.28 (0.22–0.39)	0.636
Aortic Insufficiency n, (%)					0.554
None	27 (10.0)	21 (8.6)	42 (14.5)	33 (13.3)	
Trace/Trivial	28 (10.4)	28 (11.5)	25 (8.7)	36 (14.5)	
Mild	45 (16.7)	38 (15.6)	56 (19.4)	42 (16.9)	
Moderate	60 (22.3)	49 (20.2)	54 (18.7)	41 (16.5)	
Moderate – Severe	38 (14.1)	33 (13.6)	49 (17.0)	45 (18.1)	
Severe	61 (22.7)	65 (26.7)	55 (19.0)	45 (18.1)	

Baseline demographics, comorbidities, clinical scores, investigations, indications and echocardiographic features of the study cohort, summarised by antithrombotic therapy group. Clinical Frailty Score (CFS) assessed using the Rockwood scale, ranging from 1 (very fit) to 9 (terminally ill). Higher scores indicate greater frailty. Continuous variables are presented as mean (standard deviation) or median (interquartile range), as appropriate according to distribution. Abbreviations: *Dias,* diastolic; *LV,* left ventricle; *NYHA,* New York Heart Association functional class; *PCI,* percutaneous coronary intervention; *STS,* Society of Thoracic Surgeons risk score; *Syst,* systolic

Surgical bioprosthetic valve sizes varied across groups, with sizes 23 and 25 mm most common. The Trifecta valve was the most frequently treated valve overall, followed by the Perimount and Mosaic valves (Supplementary Table 1). TAVI valves used for failed bioprosthetic valves were predominantly sizes 23- and 26-mm. Medtronic valves were most commonly implanted across all groups, followed by Edwards and Abbott. Boston Scientific valves were rarely used. (Supplementary Table 2).

At 12 months, clinical follow-up was available for nearly 90% of the patients, while echocardiographic follow-up was only available for 52% of the patients.

### 30-Day Clinical Outcomes

All-cause mortality at 30 days was significantly more frequent in the OAC (1.7%) and combination therapy (3.6%) groups compared with SAPT and DAPT (both 0.0%; *p* < 0.001) (Table 2). Life-threatening or major bleeding events were infrequent across all treatment groups: 6 (2.2%) in SAPT, 7 (2.9%) in DAPT, 6 (2.1%) in OAC, and 12 (4.8%) in the combination group. This difference was not statistically significant (*p* = 0.277). Stroke/TIA within 30 days occurred in 4 (1.5%) of SAPT, 2 (0.8%) of DAPT, 7 (2.4%) of OAC, and 4 (1.6%) of patients in the combination therapy, with no significant difference between groups (*p* = 0.574).

**Table 2:** Death, stroke/TIA and bleeding within 30 days and 12 months

	Single antiplatelet	Dual antiplatelet	Anticoagulation	Combination therapy	*P* value
Within 30 days					
Death	0/269	0/243	5/289	9/249	< 0.001
Stroke / TIA	4/269	2/243	7/289	4/249	0.574
Bleeding	6/269	7/243	6/289	12/249	0.277
MACCE	4/269	1/243	13/289	11/249	0.003
At 12 months					
Death	5/269	6/243	15/289	11/249	0.116
Stroke / TIA	0/249	0/234	1/268	1/232	0.732
Bleeding	1/249	0/234	2/268	0/232	0.625
MACCE	10/199	6/190	26/220	21/196	0.001

Incidence of death, stroke, major bleeding and MACCE within 30 days and at 12 months of valve-in-valve transcatheter aortic valve implantation, expressed as a proportion of patients with complete follow-up. Abbreviations: *MACCE,* major adverse cardiovascular and cerebrovascular events; *TIA*, transient ischaemic attack

The highest rate of MACCE was observed in the OAC (4.5%) and combination therapy (4.4%) groups, and the lowest in SAPT (1.5%) and DAPT (0.4%) (*p* = 0.003).

### 30-Day Echocardiographic Outcomes

Regarding valve haemodynamics, median aortic valve peak velocity was higher in the SAPT group compared with the OAC group (2.7 vs. 2.5 m/s; overall *p* = 0.002). Median transvalvular gradient was significantly higher in the SAPT group compared with the OAC group (17 vs. 13 mmHg; overall *p* < 0.001) (Table 3). Moderate to severe aortic insufficiency was rare, precluding meaningful statistical comparison.

**Table 3:** Post procedure day 1, day 30 and 12-month echocardiographic data

	Single antiplatelet (*n* = 269)	Dual antiplatelet (*n* = 243)	Anticoagulation (*n* = 289)	Combination therapy (*n* = 249)	*P* value
Day 1					
Ejection Fraction, mean (%) (SD)	55 (12.0)	54 (13.7)	53 (12.6)	54 (12.1)	0.205
Aortic Valve Peak Velocity, median (IQR), m/s	2.6 (2.2–3.0)	2.53 (2.2–3.0)	2.42 (2.02–2.9)	2.5 (2.09–2.9)	0.027
Aortic Valve Mean Gradient, median (IQR), mmHg	15 (10–21)	14 (10–19)	13 (9–18)	13 (9–18)	0.022
Aortic Valve Area, median (IQR), cm^2^	1.5 (1.2–1.9)	1.5 (1.2–1.9)	1.6 (1.3–2.0)	1.6 (1.28–1.9)	0.266
Aortic Insufficiency, n (%)					0.297
None	221 (82.2)	200 (82.3)	231 (79.9)	192 (77.1)	
Trace/Trivial	25 (9.3)	26 (10.7)	33 (11.4)	32 (12.9)	
Mild	11 (4.1)	7 (2.9)	14 (4.8)	11 (4.4)	
Moderate	1 (0.4)	2 (0.8)	2 (0.7)	1 (0.4)	
Moderate – Severe	0 (0.0)	0 (0.0)	0 (0.0)	0 (0.0)	
Severe	0 (0.0)	1 (0.4)	1 (0.3)	0 (0.0)	
No Data	11 (4.1)	7 (2.9)	8 (2.8)	13 (5.2)	
Paravalvular Regurgitation, n (%)					0.496
None	150 (55.8)	112 (46.1)	152 (52.6)	122 (49.0)	
Trace/Trivial	62 (23.0)	79 (32.5)	87 (30.1)	69 (27.7)	
Mild	38 (14.1)	37 (15.2)	31 (10.7)	32 (12.9)	
Moderate	7 (2.6)	4 (1.6)	7 (2.4)	9 (3.6)	
Moderate – Severe	1 (0.4)	0 (0.0)	0 (0.0)	1 (0.4)	
Severe	0 (0.0)	0 (0.0)	0 (0.0)	0 (0.0)	
No Data	11 (4.1)	11 (4.5)	12 (4.2)	16 (6.4)	
Day 30*	226 (84.0%)	204 (84.0%)	237 (82.0%)	209 (83.9%)	
Ejection Fraction, mean (%) (SD)	56.8 (9.4)	56.1 (13)	54 (10.7)	53.5 (12.4)	0.349
Aortic Valve Peak Velocity, median (IQR), m/s	2.7 (2.3–3.1)	2.5 (2.2–3.0)	2.5 (2.1–2.9)	2.5 (2.1–2.9)	0.002
Aortic Valve Mean Gradient, median (IQR), mmHg	17 (12–24)	14 (11–21)	13 (9–19)	14 (10–19)	< 0.001
Aortic Valve Area, median (IQR), cm^2^	1.4 (1.1–1.7)	1.4 (1.08–1.9)	1.5 (1.2–1.8)	1.5 (1.1–1.8)	0.189
Valvular Aortic Insufficiency, n (%)					0.012
None	177 (65.8)	152 (62.6)	168 (58.1)	155 (62.2)	
Trace/Trivial	27 (10.0)	25 (10.3)	39 (13.5)	25 (10.0)	
Mild	5 (1.9)	16 (6.6)	18 (6.2)	10 (4.0)	
Moderate	1 (0.4)	1 (0.4)	0 (0.0)	3 (1.2)	
Moderate – Severe	0 (0.0)	0 (0.0)	1 (0.3)	0 (0.0)	
Severe	0 (0.0)	0 (0.0)	0 (0.0)	0 (0.0)	
No Data	59 (21.9)	49 (20.2)	63 (21.8)	56 (22.5)	
Paravalvular Regurgitation, n (%)					0.914
None	127 (47.2)	110 (45.3)	153 (52.9)	113 (45.4)	
Trace/Trivial	50 (18.6)	40 (16.5)	50 (17.3)	48 (19.3)	
Mild	21 (7.8)	34 (14.0)	16 (5.5)	26 (10.4)	
Moderate	6 (2.2)	6 (2.5)	4 (1.4)	4 (1.6)	
Moderate – Severe	0 (0.0)	1 (0.4)	0 (0.0)	1 (0.4)	
Severe	1 (0.4)	1 (0.4)	0 (0.0)	0 (0.0)	
No Data	64 (23.8)	51 (21.0)	66 (22.8)	57 (22.9)	
12 Month †	**142 (52.8%)**	**122 (50.2%)**	**152 (52.6%)**	**136 (54.6%)**	
Ejection Fraction, mean (%) (SD)	58 (9.4)	58.7 (10.5)	56 (10.4)	56.6 (9.8)	0.221
Aortic Valve Peak Velocity, median (IQR), m/s	2.6 (2.1–3.0)	2.5 (2.1–3.0)	2.5 (2.1–2.8)	2.5 (2.1–2.9)	0.665
Aortic Valve Mean Gradient, median (IQR), mmHg	15 (10–21)	14.5 (10–20)	14 (10–18)	13 (9–19)	0.147
Aortic Valve Area, median (IQR), cm^2^	1.43 (1.1–1.9)	1.5 (1.1–1.9)	1.5 (1.2–1.96)	1.5 (1.2–1.9)	0.793
Valvular Aortic Insufficiency, n (%)					0.099
None	125 (46.5)	106 (43.6)	123 (42.6)	113 (45.4)	
Trace/Trivial	14 (5.2)	27 (11.1)	26 (9.0)	20 (8.0)	
Mild	7 (2.6)	5 (2.1)	12 (4.2)	12 (4.8)	
Moderate	2 (0.7)	4 (1.6)	1 (0.3)	1 (0.4)	
Moderate – Severe	0 (0.0)	0 (0.0)		0 (0.0)	
Severe	0 (0.0)	0 (0.0)	0 (0.0)	0 (0.0)	
No Data	121 (45.0)	101 (41.6)	127 (43.9)	103 (41.4)	
Paravalvular Regurgitation, n (%)					0.973
None	99 (36.8)	88 (36.2)	111 (38.4)	95 (38.2)	
Trace/Trivial	23 (8.6)	28 (11.5)	26 (9.0)	33 (13.3)	
Mild	20 (7.4)	17 (7.0)	14 (4.8)	18 (7.2)	
Moderate	4 (1.5)	6 (2.5)	7 (2.4)	2 (0.8)	
Moderate – Severe	1 (0.4)	0 (0.0)	1 (0.3)	0 (0.0)	
Severe	0 (0.0)	0 (0.0)	1 (0.3)	0 (0.0)	
No Data	122 (45.4)	104 (42.8)	129 (44.6)	101 (40.6)	

Serial echocardiographic haemodynamic measurements on Day 1, Day 30 and at 12 months after valve-in-valve transcatheter aortic valve implantation across the antithrombotic groups. *Data was available at 30 days for 83.5% of patients † Data was available at 12 months for 52.5% of patients

### 12-Month Clinical Outcomes

The overall mortality at 12 months did not differ significantly between groups (*p* = 0.116), with the highest rates observed in the OAC (5.2%) and combination therapy (4.4%) groups, compared to SAPT (1.8%) and DAPT (2.4%). At 12 months, life-threatening or major bleeding events and stroke/TIA rates remained low across all antithrombotic groups. The OAC group had the highest 12-month MACCE rate at 11.8%, followed by the combination therapy group at 10.7%. Both the SAPT and DAPT groups had much lower MACCE rates (5.0% and 3.2%, respectively). These differences were statistically significant (*p* = 0.001) (Table 2).

### 12-Month Echocardiographic Outcomes

At 12 months post-TAVI, there were no significant differences in left ventricular ejection fraction, aortic valve peak velocity, transvalvular gradient, or aortic valve area between antithrombotic groups (Table 3). Limited data on aortic regurgitation precluded formal statistical analysis.

### Type of TAVI Device

We assessed the impact of self-expanding valves on 30-day and 12-month mean gradients and 30-day peak velocity and whether this contributed to differences observed between treatment groups. The analysis demonstrated that self-expanding valves were associated with a mean gradient approximately 4 mmHg lower than that of balloon-expandable valves (*p* = 0.002), independent of treatment group at 12 months. However, the interaction between treatment group and valve type was not significant, indicating that the effect of self-expanding valves on mean gradient did not differ meaningfully across treatment groups (p = NS for all groups) (Supplementary Tables 3–5). Similarly, analyses evaluating the interaction between treatment group and valve size were not significant.

### Multivariable Analysis

Multivariable analysis of 30-day MACCE demonstrated higher point estimates for both OAC (OR 3.71, 95% CI 0.82–16.88; *p* = 0.090) and combination therapy (OR 2.95, 95% CI 0.70–12.40; *p* = 0.139) compared with SAPT; however, confidence intervals were wide and neither association reached statistical significance. STS score (OR 1.05 per unit increase, 95% CI 1.02–1.08; *p* = 0.004) and Rockwood Clinical Frailty Scale score (OR 1.49 per unit increase, 95% CI 1.02–2.16; *p* = 0.038) were independently associated with 30-day MACCE (Supplementary Table 6). The 30-day MACCE model demonstrated good calibration (Hosmer–Lemeshow *p* = 0.956) and good discrimination (C-statistic 0.775).

**Fig. 1:**
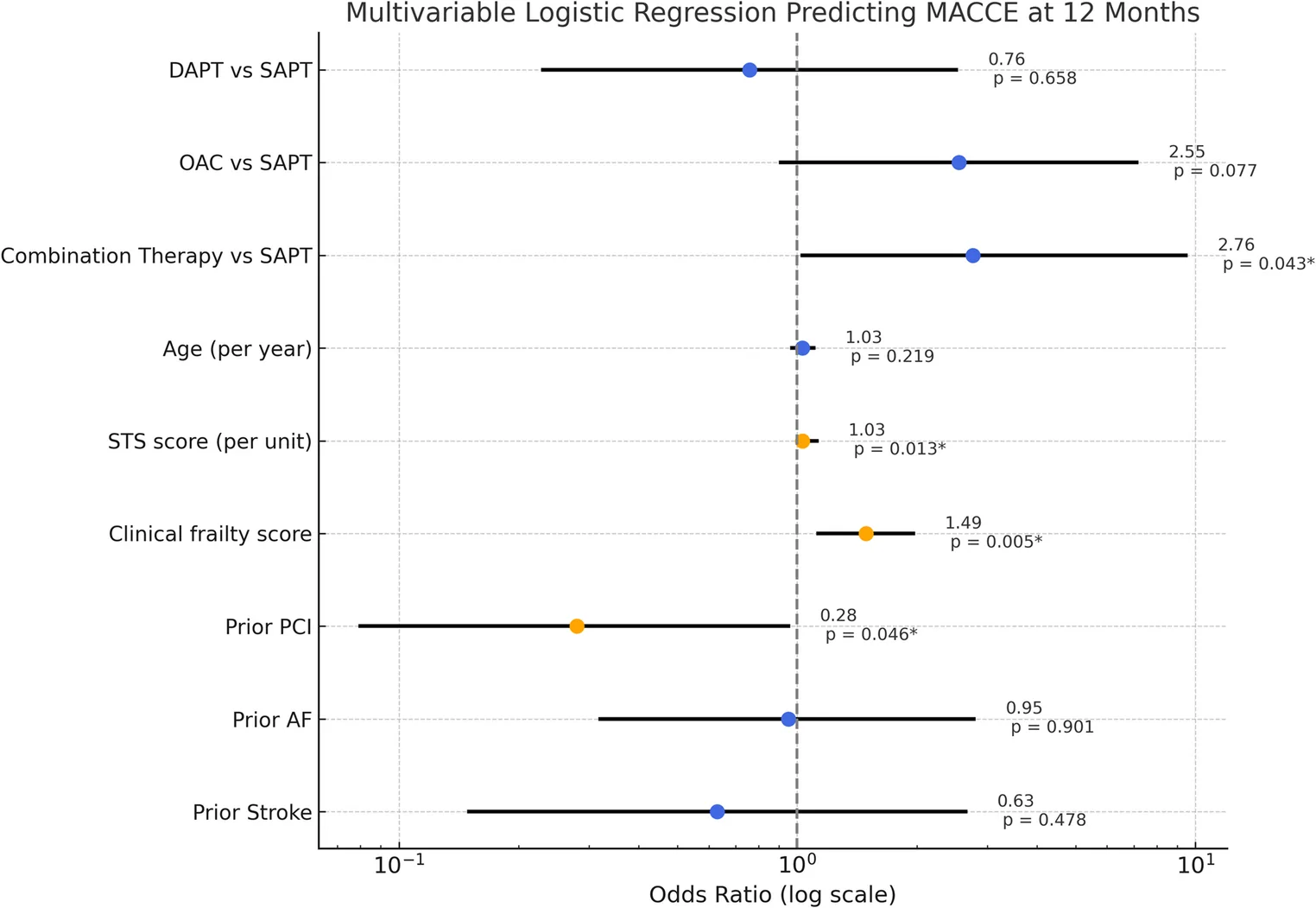
Multivariable predictors of MACCE at 12 months: forest plot of the adjusted odds ratio (OR), 95% confidence interval (horizontal bars) and p-value derived from a multivariate analysis of the predictors of MACCE at 12 months in this cohort of patients who underwent ViV TAVI for treatment of degenerated bioprosthetic aortic valves. Dashed line represents an OR of 1, and variables plotted in orange met statistical significance (*p* < 0.05). Antithrombotic regimens are compared with SAPT as the reference category. Abbreviations: AF = atrial fibrillation; Clinical Frailty Score = Rockwood Clinical Frailty Scale; DAPT = dual antiplatelet therapy; MACCE = major adverse cardiovascular and cerebrovascular events; OAC = oral anticoagulation; PCI = percutaneous coronary intervention; SAPT = single antiplatelet therapy; STS = Society of Thoracic Surgeons risk score

In the multivariable analysis of 12-month MACCE, combination therapy was independently associated with higher odds of MACCE compared with SAPT (OR 2.76, 95% CI 1.03–7.37; *p* = 0.043). The OAC group also demonstrated higher point estimates for MACCE (OR 2.55, 95% CI 0.90–7.22); however, this association did not reach statistical significance (*p* = 0.077). Given the modest number of events and wide confidence intervals, these findings should be interpreted cautiously and regarded as exploratory and hypothesis-generating. STS score (OR 1.03 per unit increase, 95% CI 1.01–1.06; *p* = 0.013) and Rockwood Clinical Frailty Scale score (OR 1.49 per unit increase, 95% CI 1.13–1.96; *p* = 0.005) were independently associated with higher odds of MACCE. In contrast, a history of prior PCI was associated with lower odds of MACCE (OR 0.28, 95% CI 0.08–0.98; *p* = 0.046) (Fig. 1; Supplementary Table 7). The 12-month MACCE model demonstrated acceptable calibration (Hosmer–Lemeshow *p* = 0.169) and discrimination (C-statistic 0.754).

No significant interaction was observed between antithrombotic regimen and baseline atrial fibrillation status for the 12-month MACCE outcome (interaction *p* = 0.563). However, given the relatively modest number of events, the analysis may have been underpowered to detect treatment-effect heterogeneity according to atrial fibrillation status.

To assess the robustness of the findings, a sensitivity analysis using a more parsimonious adjustment model including only age, STS score, and Rockwood Clinical Frailty Scale score was performed. Treatment effect estimates were materially unchanged compared with the primary model. Compared with SAPT, the odds ratio for OAC was 2.29 (95% CI 0.91–5.76; *p* = 0.077) and for combination therapy was 2.25 (95% CI 0.89–5.71; *p* = 0.088), supporting the overall consistency of the primary findings (Supplementary Table 8).

### Propensity-Weighted Analysis of Echocardiographic Outcomes

Inverse probability weighted regression adjustment (IPWRA) was performed as a sensitivity analysis for echocardiographic outcomes at 12 months. No significant differences were observed between antithrombotic groups for aortic valve area or peak aortic valve velocity after adjustment for baseline echocardiographic measurements and relevant clinical covariates (Supplementary Table 9). For mean transvalvular gradient, combination therapy showed a lower adjusted gradient compared with SAPT (ATE − 1.88 mmHg, 95% CI − 3.72 to − 0.04; unadjusted *p* = 0.046); however, this did not remain statistically significant after Bonferroni correction for multiple comparisons.

### Propensity-Weighted Analysis of Clinical Outcomes

In a propensity-weighted sensitivity analysis using inverse probability weighted regression adjustment (IPWRA), covariate balance improved across treatment groups following weighting, with reductions in standardised differences and improved variance ratios for most baseline covariates (Supplementary Tables 10 and 11). The propensity-weighted estimates were broadly consistent with the primary multivariable analysis. Compared with SAPT, no significant difference in 12-month MACCE was observed with DAPT (ATE − 1.8%, 95% CI − 5.2% to 1.6%; *p* = 0.294). OAC was associated with a numerically higher adjusted probability of MACCE (ATE 6.1%, 95% CI − 0.1% to 12.2%; *p* = 0.052), although this did not reach statistical significance. Combination therapy was associated with a significantly higher adjusted probability of MACCE compared with SAPT (ATE 7.0%, 95% CI 1.3% to 12.6%; *p* = 0.016).

## Discussion

When considering antithrombotic therapy in patients undergoing ViV TAVI, three key clinical concerns must be addressed: the risk of stroke/TIA, the occurrence of valve thrombosis (either clinical or subclinical), and the potential for bleeding complications. Each of these events is clinically important, forms part of the broader MACCE composite, and is associated with worse overall outcomes. Subclinical valve thrombosis, in particular, typically presents early after TAVI and may be identified by elevated transvalvular gradients or imaging evidence of hypoattenuated leaflet thickening (HALT).

In our study, patients receiving combination therapy or OAC demonstrated lower mean transvalvular gradients at 30 days compared to SAPT, though this difference was not sustained at 12 months. While this early haemodynamic difference may suggest less thrombotic burden in OAC-containing groups, this interpretation is speculative in the absence of cardiac CT imaging and direct HALT assessment. Furthermore, device-related factors, specifically the independent association of self-expanding valves with lower mean gradients may contribute to the observed differences and cannot be fully excluded as a confounding influence. We assessed an interaction between valve size and valve type (specifically self-expanding valves) by evaluating the p-value for interaction. Whilst the interaction term was not statistically significant, self-expanding valves were independently associated with lower mean gradients, and residual confounding in haemodynamic comparisons across antithrombotic groups remains plausible.

The haemodynamic performance of currently available transcatheter heart valves warrants consideration when interpreting gradient differences across antithrombotic groups. Self-expanding valves have generally been associated with lower mean transvalvular gradients compared to balloon-expandable valves, and in the ViV setting these device-specific characteristics may be further influenced by the constrained geometry of the surgical valve frame. As highlighted by Angellotti et al., interpretation of haemodynamic parameters should account for prosthesis type, size, and implant position, and it is possible that the distribution of valve platforms across antithrombotic groups contributed in part to the observed gradient differences [14]. 

Unfortunately, we did not have access to cardiac computed tomography (CT) analysis to assess hypo-attenuated leaflet thickening (HALT) in this cohort. It is important to recognise that subclinical leaflet thrombosis encompasses a spectrum of severity. HALT represents the milder form and may not significantly affect transvalvular gradients, as demonstrated by Garcia et al. [15–17] In contrast, reduced leaflet motion (RELM), which occurs secondary to HALT and reflects a higher thrombotic burden, is associated with more significant haemodynamic impairment [18]. Standardised CT methodology for the assessment of HALT and RELM has been well described, and expert consensus guidance now exists to guide their clinical evaluation [19, 20]. In the absence of CT imaging in our cohort, we are unable to distinguish between these phenotypes or determine whether the early gradient differences observed reflect HALT, RELM, or neither. While a modest improvement in transvalvular gradient was observed in the anticoagulation group, the long-term impact on valve performance may be more substantial than early gradient measurements suggest. This is because the presence of HALT in the early postoperative period does not necessarily correlate with immediate increases in gradients, and thus, its clinical consequences may not be fully appreciated in the short term.

The early improvements in valve haemodynamics observed on echocardiography were not sustained at the 12-month follow-up. Several factors may account for this observation, including the relatively short duration of follow-up, a limited sample size, variability in the timing of echocardiographic assessments at 12 months, or the inherently dynamic nature of HALT. Hypo-attenuated leaflet thickening is known to be a reversible phenomenon that may both develop and regress spontaneously over time. This concept is supported by data from the PARTNER 3 sub-study by Makkar et al., which demonstrated that HALT identified at 30 days had spontaneously resolved in 54% of patients by the 1-year mark [21]. These findings highlight the transient nature of HALT and underscore the importance of longitudinal imaging and follow-up when assessing its clinical significance and impact on valve function.

Despite the early haemodynamic differences observed with OAC, our findings raise important clinical questions regarding long-term outcomes. At 30 days, mortality was higher among patients receiving any form of OAC—either as monotherapy or in combination with antiplatelet therapy—compared to those treated with single or dual antiplatelet therapy, though this observation must be interpreted in the context of substantial baseline differences between groups. MACCE rates were higher at 30 days and 12 months among patients in the OAC and combination therapy groups.

It is important to emphasise that this was not a randomised study, and OAC was prescribed based on established clinical indications, most notably atrial fibrillation. The most likely drivers of the observed differences in mortality and MACCE are the substantially higher prevalence of atrial fibrillation, greater STS scores, heart failure, and chronic kidney disease in the OAC and combination therapy groups, all of which are independently associated with worse cardiovascular outcomes. To further explore this association, we conducted a multivariable logistic regression analysis focused on MACCE. This analysis identified combination therapy as independently associated with higher 12-month MACCE, though residual confounding cannot be excluded given the observational nature of the study. Additionally, a signal was observed suggesting a higher MACCE with OAC monotherapy, although this did not reach a statistically significant level. These findings should be considered exploratory and hypothesis-generating and warrant confirmation in prospective studies specifically designed to evaluate antithrombotic strategies following ViV TAVI. Notably, the lower MACCE observed in the DAPT group may in part reflect the higher prevalence of prior PCI in this cohort, which was an independent predictor of lower MACCE on multivariable analysis. This is consistent with recent randomised evidence from the NOTION-3 and FAITAVI trials demonstrating that coronary revascularisation with PCI reduces adverse cardiac events in patients undergoing TAVI with concomitant coronary artery disease [22, 23]. 

The observation of a very low incidence of 12-month bleeding events in the combination therapy group was unexpected and should not be interpreted as evidence that OAC plus antiplatelet therapy is inherently safe from a bleeding standpoint. Three potential explanations may account for this finding. First, the definition of major bleeding used in the study was stringent, requiring severe or catastrophic events to meet the criteria; thus, less severe but clinically relevant bleeding episodes may have gone unreported. Second, in the combination therapy group, antiplatelet therapy is typically discontinued after six months, which could have contributed to a reduced risk of bleeding in the later follow-up period. Third, the overall follow-up rate at 12 months was not complete. Patients who were lost to follow-up, declined participation, or were otherwise unreachable might have experienced significant bleeding events that were not captured in the final analysis. Consequently, the low bleeding rate observed may reflect a combination of underreporting due to strict criteria, reduced antiplatelet exposure over time, and incomplete data capture.

Several limitations of this study should be acknowledged. First, as a non-randomised observational analysis, treatment allocation was not standardised and may have introduced selection bias and confounding by indication, particularly as OAC was more frequently prescribed to patients with atrial fibrillation and a greater burden of comorbidities. Although multivariable adjustment, parsimonious sensitivity analyses, and propensity-weighted analyses were performed, residual confounding cannot be excluded.

Second, outcome ascertainment was constrained by the structure of the registry. Exact dates of clinical events were not available within the registry extract, and outcomes were recorded at predefined follow-up intervals (30 days and 12 months). Consequently, time-to-event analyses such as Cox proportional hazards or Fine–Gray competing risk models could not be reliably performed, and outcomes were analysed as binary events at fixed follow-up time points. Although clinical follow-up at 12 months was available for approximately 90% of patients, this approach does not account for event timing and may be susceptible to bias in the presence of incomplete follow-up. Furthermore, the number of clinical events was modest relative to the number of covariates included in some multivariable models, increasing the risk of overfitting, unstable estimates, and wide confidence intervals. Accordingly, adjusted associations, particularly those close to the threshold of statistical significance, should be interpreted cautiously and regarded as hypothesis-generating.

Third, assessment of valve haemodynamics was limited by incomplete imaging follow-up and the absence of advanced valve imaging. Echocardiographic follow-up at 12 months was available in only 52% of patients, potentially introducing bias into haemodynamic analyses. In addition, the lack of cardiac computed tomography precluded assessment of hypo-attenuated leaflet thickening (HALT) and reduced leaflet motion, limiting our ability to directly evaluate subclinical leaflet thrombosis or correlate imaging findings with clinical outcomes.

Finally, several important variables were not captured within the ACOR TAVI Registry. The registry does not record the specific indication for OAC or antiplatelet therapy, the proportion of vitamin K antagonists versus direct oral anticoagulants, the duration of concomitant antiplatelet therapy, or changes in antithrombotic treatment during follow-up. As such, the antithrombotic categories used in this analysis represent a necessarily broad exposure classification. The combination therapy group in particular likely reflects a clinically heterogeneous population, including patients with atrial fibrillation and concomitant coronary artery disease, recent PCI, or other complex vascular phenotypes, rather than a uniform treatment strategy. In addition, true internal diameter, valve fracture, prosthesis–patient mismatch, and other detailed prosthesis-related characteristics were not available, precluding complete adjustment for prosthesis-related confounding in haemodynamic comparisons. Bleeding events may also have been underestimated because only VARC-3 life-threatening or major bleeding events were captured and follow-up was incomplete. Taken together, these limitations highlight the need for prospective, randomised studies specifically designed to evaluate antithrombotic strategies in patients undergoing ViV TAVI.

In conclusion, discharge antithrombotic regimen was associated with differences in clinical outcomes and early valve haemodynamics following ViV TAVI. OAC-containing regimens were associated with higher rates of MACCE, while lower transvalvular gradients were observed at 30 days. However, given the observational nature of the study and the potential for residual confounding by indication, these findings should be interpreted cautiously and not as evidence of causality. Until randomised data become available, antithrombotic therapy following ViV TAVI should remain individualised, particularly in patients without an established indication for long-term anticoagulation.

## Supplementary Information

Below is the link to the electronic supplementary material.

Supplementary Material 1

## Data Availability

The data that support the findings of this study were obtained from the Australasian Cardiac Outcomes Registry (ACOR) TAVI/ATVT Registry. These data are not publicly available because access is governed by ACOR and subject to data governance, ethics, and privacy restrictions. De-identified data may be made available to eligible researchers on reasonable request and with permission from ACOR, following review by the ACOR Research & Publications Committee and any required institutional ethics approvals.
