# Supplementary material for: Comparative Analysis of Antithrombotic Strategies Following Valve-in-Valve Transcatheter Aortic Valve Implantation Using Real-World Registry Data
Source: Cardiovasc Drugs Ther. 2026 Jul 17;40(6):2303–14. doi: 10.1007/s10557-026-07914-2 (PMC13748142; doi:10.1007/s10557-026-07914-2)
Supplement: Supplementary file 1 [file 10557_2026_7914_MOESM1_ESM.docx]

**Supplementary Table 1: Baseline Bioprosthetic Valve Details**

|  | Single Antiplatelet (n=269) | Dual Antiplatelet  (n=243) | Anticoagulation  (n=289) | Combination Therapy (n=249) |
| --- | --- | --- | --- | --- |
|  |  |  |  |  |
| Valve Size (mm) (%) |  |  |  |  |
| 19 | 7 (2.6) | 4 (1.6) | 8 (2.8) | 5 (2.0) |
| 21 | 28 (10.4) | 29 (11.9) | 40 (13.7) | 39 (15.6) |
| 23 | 72 (26.7) | 44 (18.0) | 64 (22) | 56 (22.4) |
| 25 | 79 (29.3) | 64 (26.2) | 64 (22.1) | 55 (22.0) |
| 27 | 27 (10.0) | 25 (10.2) | 42 (14.4) | 22 (8.8) |
| 29 | 16 (6.0) | 7 (2.9) | 20 (6.9) | 16 (6.4) |
| No Data | 40 (14.8) | 70 (28.7) | 51 (17.6) | 55 (22.0) |
| Bioprosthetic Valve Model, n (%) |  |  |  |  |
| 3F Valve |  |  |  | 1 (0.4) |
| Biocor/Epic | 8 (3.0) | 5 (2.1) | 4 (1.4) | 2 (0.8) |
| Biocor/Epic Supra |  |  |  | 1 (0.4) |
| Biovalsalva Porcine Conduit |  |  | 1 (0.3) |  |
| CE SAV | 5 (1.9) | 2 (0.8) | 1 (0.3) | 2 (0.8) |
| CE Standard 2625 |  | 1 (0.4) |  | 1 (0.4) |
| Freedom Solo | 1 (0.4) |  |  |  |
| Freestyle Root | 4 (1.5) | 2 (0.8) | 2 (0.7) |  |
| Freestyle Valve | 6 (2.2) | 2 (0.8) | 1 (0.3) | 2 (0.8) |
| Hancock II | 1 (0.4) |  | 1 (0.3) |  |
| Inspiris Resilia |  |  |  | 1 (0.4) |
| Intuity |  |  | 3 (1.0) | 1 (0.4) |
| Magna 3000 | 4 (1.5) | 4 (1.6) | 9 (3.1) | 2 (0.8) |
| Magna Ease 3300 | 16 (5.9) | 17 (7.0) | 20 (6.9) | 18 (7.2) |
| Mitroflow | 12 (4.5) | 23 (9.5) | 22 (7.6) | 17 (6.8) |
| Mosaic | 25 (9.3) | 27 (11.1) | 31 (10.7) | 18 (7.2) |
| Perceval | 9 (3.3) | 8 (3.3) | 8 (2.8) | 11 (4.4) |
| Perimount 2700 | 36 (13.4) | 23 (9.5) | 29 (10.0) | 35 (14.1) |
| Perimount 2800 | 8 (3.0) | 17 (7.0) | 28 (9.7) | 15 (6.0) |
| Torento SPV Root | 1 (0.4) |  |  |  |
| Trifecta | 50 (18.6) | 29 (11.9) | 41 (14.2) | 29 (11.6) |
| Trifecta GT |  |  |  | 1 (0.4) |
| Other | 45 (16.7) | 17 (7.0) | 46 (15.9) | 35 (14.1) |
| No Data | 38 (14.1) | 66 (27.2) | 42 (14.5) | 57 (22.9) |

Characteristics of the failed bioprosthetic surgical valves prior to valve in valve transcatheter aortic valve implantation, including brand, size and mode of degeneration.

**Supplementary Table 2: Valve-in-Valve Transcatheter Aortic Valve Implant Details**

|  | Single Antiplatelet(n=269) | Dual Antiplatelet  (n=243) | Anticoagulation  (n=289) | Combination Therapy (n=249) |
| --- | --- | --- | --- | --- |
|  |  |  |  |  |
| Valve Size (mm) (%) |  |  |  |  |
| 20 | 6 (2.2) | 3 (1.2) | 5 (1.7) | 7 (2.8) |
| 23 | 101 (37.5) | 94 (38.7) | 115 (39.8) | 94 (37.8) |
| 25 | 13 (4.8) | 10 (4.1) | 12 (4.2) | 3 (1.2) |
| 26 | 98 (36.4) | 88 (36.2) | 94 (32.5) | 89 (35.7) |
| 27 | 3 (1.1) | 3 (1.2) | 12 (4.2) | 4 (1.6) |
| 29 | 35 (13) | 32 (13.2) | 36 (12.5) | 41 (16.5) |
| 34 | 7 (2.6) | 6 (2.5) | 8 (2.8) | 5 (2.0) |
| Valve Manufacturer, n (%) |  |  |  |  |
| Edwards | 113 (42.0) | 61 (25.2) | 96 (33.2) | 90 (36.1) |
| Medtronic | 121 (45.0) | 156 (64.5) | 150 (51.9) | 136 (54.6) |
| Abbott | 32 (11.9) | 25 (10.3) | 40 (13.8) | 20 (8.0) |
| Boston Scientific | 0 | 0 | 0 | 1 (0.4) |

Procedural details of valve-in-valve transcatheter aortic valve implant, including valve manufacturer and valve size by antithrombotic group.

**Supplementary Table 3: P-values for interaction between type of TAVI device and antithrombotic treatment group on aortic valve mean gradient at 30 days**

| **Variable** | **Co-efficient (mmHg) (95% CI)** | **Std. Err.** | **t** | **p-value** | **95% CI** |
| --- | --- | --- | --- | --- | --- |
| DAPT | −0.30 | 1.39 | −0.22 | 0.830 | (−3.02, 2.43) |
| OAC | **−4.14** | 1.25 | −3.31 | **0.001** | (−6.60, −1.69) |
| Combination Therapy | **−3.14** | 1.28 | −2.45 | **0.015** | (−5.66, −0.62) |
| **Self-expanding valve** | **−5.11** | 1.13 | −4.50 | **<0.001** | (−7.33, −2.88) |
| **Interaction terms (group#selfexpa)** |  |  |  |  |  |
| DAPT#1 | −1.47 | 1.73 | −0.85 | 0.398 | (−4.87, 1.94) |
| OAC#1 | 1.70 | 1.62 | 1.05 | 0.294 | (−1.48, 4.89) |
| Other#1 | 0.08 | 1.68 | 0.05 | 0.960 | (−3.21, 3.37) |
| **_cons (intercept)** | 21.32 | 0.84 | 25.33 | <0.001 | (19.66, 22.97) |

Interaction analysis showing mean transvalvular gradient at 30 days stratified by valve type (self-expanding expanding VS balloon expandable) and antithrombotic regimen (as compared to single antiplatelet therapy). Abbreviations: DAPT = dual antiplatelet therapy; OAC = oral anticoagulation; Std. Err = standard error; t = t-statistic; TAVI = transcatheter aortic valve implantation.

**Supplementary Table 4: P-values for interaction between type of TAVI device and antithrombotic treatment group on aortic valve peak velocity at 30 days**

| **Variable** | **Co-efficient (m/s) (95% CI)** | **Std. Err.** | **t** | **P value** | **95% CI** |
| --- | --- | --- | --- | --- | --- |
| DAPT | −0.06 | 0.11 | −0.56 | 0.573 | (−0.28, 0.15) |
| OAC | **−0.27** | 0.10 | −2.75 | **0.006** | (−0.46, −0.08) |
| Combination Therapy | −0.17 | 0.10 | −1.67 | 0.096 | (−0.36, 0.03) |
| **Self-expanding valve (1 = yes)** | **−0.35** | 0.09 | −3.89 | **<0.001** | (−0.52, −0.17) |
| **Interaction terms (group#selfexpa)** |  |  |  |  |  |
| DAPT#1 | −0.04 | 0.14 | −0.26 | 0.799 | (−0.30, 0.23) |
| OAC#1 | 0.15 | 0.13 | 1.17 | 0.242 | (−0.10, 0.40) |
| Other#1 | −0.01 | 0.13 | −0.10 | 0.919 | (−0.27, 0.24) |
| **_cons (intercept)** | 2.92 | 0.07 | 43.67 | <0.001 | (2.78, 3.05) |

Interaction analysis showing aortic valve peak velocity at 30 days stratified by valve type (self-expanding expanding VS balloon expandable) and antithrombotic regimen (as compared to single antiplatelet therapy). Abbreviations: DAPT = dual antiplatelet therapy; OAC = oral anticoagulation; Std. Err = standard error; t = t-statistic; TAVI = transcatheter aortic valve implantation.

**Supplementary Table 5: P-values for interaction between type of TAVI device and antithrombotic treatment group on aortic valve mean gradient at 12 months.**

| **Variable** | **Co-efficient (mmHg) (95% CI)** | **Std. Err** | **t** | **P value** | **95% CI** |
| --- | --- | --- | --- | --- | --- |
| DAPT | 0.72 | 1.54 | 0.47 | 0.642 | (-2.31, 3.75) |
| OAC | -2.01 | 1.44 | -1.39 | 0.164 | (-4.83, 0.82) |
| Combination Therapy | -1.73 | 1.45 | -1.20 | 0.232 | (-4.57, 1.11) |
| **Self-expanding valve (1 = yes)** | -4.09 | 1.32 | -3.09 | 0.002 | (-6.68, -1.49) |
| **Interaction terms (group#selfexpa)** |  |  |  |  |  |
| DAPT#1 | -1.69 | 1.95 | -0.87 | 0.386 | (-5.51, 2.14) |
| OAC#1 | 1.35 | 1.87 | 0.72 | 0.469 | (-2.31, 5.02) |
| Other#1 | -1.14 | 1.89 | -0.60 | 0.548 | (-4.85, 2.58) |
| **_cons (intercept)** | 19.19 | 0.99 | 19.48 | 0.000 | (17.26, 21.13) |

Interaction analysis showing aortic valve mean gradient at 12 months stratified by valve type (self-expanding expanding VS balloon expandable) and antithrombotic regimen (as compared to single antiplatelet therapy). Abbreviations: DAPT = dual antiplatelet therapy; OAC = oral anticoagulation; Std. Err = standard error; t = t-statistic; TAVI = transcatheter aortic valve implantation.

**Supplementary Table 6. Multivariable Logistic Regression Analysis for 30-Day MACCE**

| Variable | OR | 95% CI | p-value |
| --- | --- | --- | --- |
| DAPT vs SAPT | 0.39 | 0.04–3.87 | 0.423 |
| OAC vs SAPT | 3.71 | 0.82–16.88 | 0.090 |
| Combination therapy vs SAPT | 2.95 | 0.70–12.40 | 0.139 |
| Age (per year) | 0.99 | 0.94–1.04 | 0.756 |
| STS score (per unit increase) | 1.05 | 1.02–1.08 | 0.004 |
| Rockwood Clinical Frailty Scale (per unit increase) | 1.49 | 1.02–2.16 | 0.038 |
| Prior atrial fibrillation | 0.59 | 0.20–1.75 | 0.344 |
| Prior stroke | 1.15 | 0.24–5.47 | 0.862 |

Multivariable Logistic Regression Analysis for 30-Day MACCE. Prior PCI was omitted from the 30-day model because of complete separation, resulting in exclusion of 113 observations. Abbreviations: SAPT, single antiplatelet therapy; DAPT, dual antiplatelet therapy; OAC, oral anticoagulation; STS, Society of Thoracic Surgeons risk score; TIA, transient ischaemic attack.

**Supplementary Table 7: Multivariable Logistic Regression Results for Predicting MACCE at 12 Months**

| **Variable** | **Odds Ratio** | **Std. Error** | **z-value** | **P value** | **95% Confidence Interval** |
| --- | --- | --- | --- | --- | --- |
| **DAPT ^t^** | 0.76 | 0.464 | -0.44 | 0.658 | 0.23 – 2.51 |
| **OAC ^t^** | 2.55 | 1.354 | 1.77 | 0.077 | 0.90 – 7.22 |
| **Combination therapy ^t^** | 2.76 | 1.383 | 2.02 | 0.043 | 1.03 – 7.37 |
| **Age (per year)** | 1.03 | 0.022 | 1.23 | 0.219 | 0.98 – 1.07 |
| **STS score (per unit)** | 1.03 | 0.014 | 2.48 | 0.013 | 1.01 – 1.06 |
| **Rockwood Clinical Frailty Scale** | 1.49 | 0.210 | 2.84 | 0.005 | 1.13 – 1.96 |
| **Prior PCI** | 0.28 | 0.179 | -1.99 | 0.046 | 0.08 – 0.98 |
| **Prior AF** | 0.95 | 0.359 | -0.12 | 0.901 | 0.46 – 1.99 |
| **Prior Stroke** | 0.63 | 0.408 | -0.71 | 0.478 | 0.18 – 2.24 |

Multivariable logistic regression identifying predictors of MACCE at 12 months following valve in valve transcatheter aortic valve implantation. **^t^**Antithrombotic reference group is Single Antiplatelet. Abbreviations: AF = atrial fibrillation; DAPT = dual antiplatelet therapy; OAC = oral anticoagulation; PCI = percutaneous coronary intervention; STS = Society of Thoracic Surgeons risk score; Std. Error = standard error; z-value = z-statistic.

**Supplementary Table 8 Sensitivity Analysis Using a Parsimonious Multivariable Model for 12-Month MACCE**

| Variable | OR | 95% CI | p-value |
| --- | --- | --- | --- |
| DAPT vs SAPT | 0.68 | 0.21–2.23 | 0.530 |
| OAC vs SAPT | 2.29 | 0.91–5.76 | 0.077 |
| Combination therapy vs SAPT | 2.25 | 0.89–5.71 | 0.088 |
| Age (per year) | 1.02 | 0.98–1.07 | 0.267 |
| STS score (per unit increase) | 1.04 | 1.01–1.06 | 0.011 |
| Clinical Frailty Score (per unit increase) | 1.46 | 1.11–1.92 | 0.007 |

Model performance: Likelihood ratio χ² = 36.17 (6 df), p < 0.001, Pseudo R² = 0.107. A parsimonious multivariable logistic regression model was constructed as a sensitivity analysis including only age, STS score, and Clinical Frailty Score. Effect estimates were materially similar to those observed in the primary adjusted model, supporting the robustness of the findings. Abbreviations: SAPT, single antiplatelet therapy; DAPT, dual antiplatelet therapy; OAC, oral anticoagulation; STS, Society of Thoracic Surgeons risk score; MACCE, major adverse cardiovascular and cerebrovascular events.

**Supplementary Table 9.** IPWRA Analysis of Echocardiographic Outcomes at 12 Months

| **Outcome** | **Comparison** | **ATE** | **95% CI** | **p** | **Bonferroni-adjusted p** |
| --- | --- | --- | --- | --- | --- |
| Aortic valve area | DAPT vs SAPT | 0.14 | -0.01 to 0.29 | 0.066 | 0.198 |
|  | OAC vs SAPT | 0.04 | -0.12 to 0.20 | 0.620 | >0.999 |
|  | Combination vs SAPT | -0.03 | -0.17 to 0.11 | 0.706 | >0.999 |
| Peak velocity | DAPT vs SAPT | -0.04 | -0.21 to 0.14 | 0.675 | >0.999 |
|  | OAC vs SAPT | -0.09 | -0.22 to 0.05 | 0.215 | 0.645 |
|  | Combination vs SAPT | -0.05 | -0.19 to 0.08 | 0.428 | >0.999 |
| Mean gradient | DAPT vs SAPT | -0.97 | -2.88 to 0.95 | 0.322 | 0.966 |
|  | OAC vs SAPT | -1.29 | -3.45 to 0.86 | 0.240 | 0.720 |
|  | Combination vs SAPT | -1.88 | -3.72 to -0.04 | 0.046 | 0.138 |

Inverse probability weighted regression adjustment (IPWRA) analysis of 12-month echocardiographic outcomes according to discharge antithrombotic regimen. Models were adjusted for the corresponding 30-day post-procedure echocardiographic measurement, age, prior percutaneous coronary intervention (PCI), atrial fibrillation, and prior stroke/TIA. Values are presented as average treatment effects (ATE) with 95% confidence intervals. Bonferroni-adjusted p-values were calculated for the three pairwise treatment comparisons within each echocardiographic outcome. Abbreviations: ATE, average treatment effect; DAPT, dual antiplatelet therapy; OAC, oral anticoagulation; SAPT, single antiplatelet therapy; PCI, percutaneous coronary intervention; TIA, transient ischaemic attack.

**Supplementary Table 10.** IPWRA. Analysis for 12-Month MACCE

| **Comparison** | **Average Treatment Effect (ATE)** | **95% CI** | **p-value** |
| --- | --- | --- | --- |
| DAPT vs SAPT | -0.018 | -0.052 to 0.016 | 0.294 |
| OAC vs SAPT | 0.061 | -0.001 to 0.122 | 0.052 |
| Combination therapy vs SAPT | 0.070 | 0.013 to 0.126 | 0.016 |

Inverse Probability Weighted Regression Adjustment (IPWRA) models were constructed to account for baseline differences between treatment groups. The treatment model included chronic kidney disease, prior acute myocardial infarction, atrial fibrillation, prior stroke, prior PCI, and prior coronary artery bypass graft surgery. The outcome model included coronary artery disease, diabetes mellitus, chronic kidney disease, STS score, Rockwood Clinical Frailty Scale score, and prior CABG. Abbreviations: ATE, average treatment effect; DAPT, dual antiplatelet therapy; MACCE, major adverse cardiac and cerebrovascular events; OAC, oral anticoagulation; SAPT, single antiplatelet therapy.

**Supplementary Table 11**. Covariate Balance Before and After Inverse Probability Weighted Regression Adjustment (IPWRA)

| **Covariate** | **Standardized Difference (Raw)** | **Standardized Difference (Weighted)** |
| --- | --- | --- |
|  |  |  |
| Chronic kidney disease | 0.04–0.28 | -0.10 to -0.06 |
| Prior PCI | -0.02–0.40 | 0.02–0.08 |
| Prior acute myocardial infarction | 0.04–0.20 | -0.10 to 0.07 |
| Atrial fibrillation | 0.01–1.67 | -0.03 to 0.02 |
| Prior stroke | 0.23–0.39 | 0.04–0.15 |
| Prior CABG | 0.11–0.36 | -0.05 to 0.02 |

Footnote: Covariate balance improved substantially after weighting, with standardized differences markedly reduced across treatment groups. Full balance diagnostics, including variance ratios, are available upon request.
